# Purchase Intention of Healthy Foods: The Determinant Role of Brand Image in the Market of a Developing Country

**DOI:** 10.3390/foods13203242

**Published:** 2024-10-12

**Authors:** Elizabeth Emperatriz García-Salirrosas, Manuel Escobar-Farfán, Iván Veas-González, Jorge Alberto Esponda-Perez, Rodrigo Gallardo-Canales, Rodrigo Ruiz-Andia, Virginia Mercedes Fernandez-Daza, Rosa Fabiana Zabalaga-Davila

**Affiliations:** 1 Grupo de Investigación e Innovación para el Emprendimiento y Sostenibilidad, Universidad Nacional Tecnológica de Lima Sur, Lima 15816, Peru; 2 Department of Administration, Faculty of Administration and Economics, University of Santiago of Chile, Santiago 9170020, Chile; manuel.escobar@usach.cl; 3 Departamento de Administración, Facultad de Economía y Administración, Universidad Católica del Norte, Antofagasta 1270709, Chile; iveas@ucn.cl; 4 Faculty of Nutrition and Food Sciences, Universidad de Ciencias y Artes de Chiapas, Tuxtla Gutiérrez 29000, Mexico; jorge.esponda@unicach.mx; 5 Department of Management Technologies, Faculty of Technology, University of Santiago of Chile, Santiago 9170020, Chile; rodrigo.gallardo@usach.cl; 6 Centro de Investigación en Ciencias Exactas e Ingenierías (CICEI), Universidad Católica Boliviana San Pablo, Cochabamba, Bolivia; rodrigo.ruiz@ucb.edu.bo (R.R.-A.); rzabalaga@ucb.edu.bo (R.F.Z.-D.); 7 Departamento de Ciencias Empresariales, Universidad del Valle, Cochabamba, Bolivia; vfernandezd@univalle.edu

**Keywords:** brand image, purchase intention, perceived brand quality, brand satisfaction, brand trust, brand loyalty, healthy foods, developing country

## Abstract

In the current consumer context, the trend towards a healthy lifestyle has significantly increased the demand for healthy foods. This study aims to identify the relationship between the brand image (BI) and purchase intention (PI) of these products and how variables such as perceived brand quality (BPQ) and brand satisfaction (BS) influence brand trust (BT) and brand loyalty (BL) in this relationship. The methodology includes a quantitative approach, using non-probability convenience sampling. Using an online survey, data were collected from 637 consumers. Analyses were performed using structural equation modeling (SEM-PLS). The results show no significant correlation between BI and PI, but BI significantly impacts BPQ, BS, BT, and BL. Furthermore, BPQ positively influences BS, BT, and BL, but it does not have a direct influence on PI. The findings suggest that a positive brand image satisfies consumers and generates long-term trust and loyalty. However, perceived quality does not always translate into purchase intention due to various barriers. Practical implications highlight the importance of building a strong and positive brand image to encourage demand for healthy products.

## 1. Introduction

Within today’s consumption contexts, the trend towards a healthy lifestyle has significantly increased the demand for healthy foods [1,2,3,4,5,6]. These foods, characterized by their nutritional value and health benefits, play a crucial role in preventing diseases and promoting a healthier life [7,8,9,10,11]. However, the choosing of these products involves an extrinsic factor, such as brand image [12]. Other factors, such as price (as consumers tend to opt for products that fit their budget [8]), food packaging [13], and other marketing strategies, such as colors and prominent information on the product label [3], all play crucial roles.

Brand image is a multifaceted concept encompassing consumers’ perceptions and feelings about a brand [14,15]. In the context of healthy foods, a positive brand image (BI) can be crucial for purchase intention (PI), as consumers tend to prefer brands that represent quality, trust, and satisfaction [16]. This encourages consumer loyalty to the brand [17,18,19]. Furthermore, brand image can indirectly influence purchase intention through other variables such as a brand’s perceived quality, brand satisfaction, brand trust, and brand loyalty [12,18,20,21]. Therefore, it is essential to understand how these factors influence the consumer’s purchasing decisions [22,23].

On the other hand, consumers’ interpretation of information is deeply influenced by psychological factors that affect their decision making. One such factor is health perception, where keywords such as “organic”, “low fat”, or “sugar-free” generate a positive impression and can lead to the choice of one product over another [2]. Another factor is familiarity and trust in a brand, where product images that evoke positive memories or are associated with previous satisfying experiences can tip the balance in favor of that choice [3]. In addition, the halo effect can play a significant role; if a product is perceived as healthy in one aspect, consumers tend to assume that it is healthy in other aspects as well, which influences their final decision [24]. These psychological factors act together to guide label interpretation and, ultimately, product selection.

BPQ refers to the consumer’s perception of a product or service’s overall quality compared to available alternatives [25,26]. In the healthy food market, perceived quality can significantly influence the purchasing decision, as consumers look for products that are not only healthy but also meet high quality standards [17,27]. In this sense, a positive perception of quality can reinforce brand image and increase purchase intention [28].

On the other hand, brand satisfaction measures how well a brand’s products or services meet or exceed consumer expectations [29]. High satisfaction can lead to increased purchase intention, as satisfied consumers repeat purchases and recommend the brand to others [30,31]. Thus, customer satisfaction bridges brand image and purchase intention [12,18,21], strengthening the relationship between both concepts [19].

Brand trust is another critical factor influencing purchase decisions [21,32]. According to Akoglu and Özbek [20], brand trust is the belief in the reliability and honesty that a brand represents in a consumer. In the context of healthy foods, BT is crucial, as consumers must ensure that the products they buy are safe and beneficial for their health [33]. In this sense, greater brand trust may increase purchase intention by reducing the perception of risk associated with purchasing healthy foods [22]. In this way, brand trust protects consumers and strengthens the relationship between brand image and purchase intention [12].

Finally, brand loyalty refers to consumers’ willingness to purchase a specific brand despite marketing influences and competitor efforts [34,35]. Brand loyalty facilitates repeat purchases and attracts new consumers through recommendations and positive opinions from those loyal to consuming a brand [36]. Therefore, brand loyalty results from a strong and positive brand image, which, in turn, reinforces purchase intention [37].

Despite the growing interest in healthy foods, there is a lack of understanding about the influence of brand image on brand perceived quality [27], brand satisfaction [31], brand trust [21], and brand loyalty [20] and how these variables then influence the PI of healthy products in developing countries. In this sense, the present study will provide valuable insights for marketers and brands seeking to capitalize on the growing interest in healthy products. In addition, it will provide important theoretical information that will improve the understanding of consumers who identify with the purchase of this type of product.

Considering the above, this research aims to answer the following research question: What is the relationship between brand image and purchase intention for healthy foods? The research sub-questions are as follows: (a) How does brand image influence brand perceived quality, brand satisfaction, brand trust, and brand loyalty? (b) How do brand perceived quality, brand satisfaction, brand trust, and brand loyalty influence the purchase intention of healthy products in a developing economy?

## 2. Literature Review

Brand image refers to consumers’ overall perception of a company, product, or service in the consumer’s mind [38,39]. This perception can induce positive or negative emotions and thoughts. It is shaped by all the experiences, interactions, and characteristics consumers associate with a brand [39,40]. A strong and appropriate brand image is not just a marketing tool but a significant influencer of consumer behavior, especially when consumers understand the health benefits associated with the product. This understanding can drive consumers to make more informed and healthy purchasing decisions [41,42].

Perceived quality refers to the quality of a product or service according to the customer’s perception [39]. It is a subjective criterion that does not have to coincide with real or objective quality. It is based on tangible data such as raw materials, the manufacturing process, warranty, and after-sales service [43,44,45]. The perceived quality of a brand in the healthy food domain is the customer’s understanding of the excellence or mediocrity of a healthy product. It is crucial to note that customer perception can vary significantly from one customer to another, adding a layer of complexity to the understanding of consumer behavior [46,47,48].

Brand satisfaction refers to the extent to which a product or service from a specific brand meets or exceeds customer expectations. It is a crucial component of brand loyalty and can influence customer purchasing behavior, including repeat purchases and recommending the brand to others [49,50]. In the context of healthy foods, it refers to how consumers evaluate their experience with a specific healthy food brand. This evaluation is based on whether the brand has met its expectations [41,51].

Brand trust is the consumer’s expectation that a product or service is reliable and will deliver on its promises [52,53]. Brand trust in food marketing is essential because brands provide concise information for consumers [54]. Therefore, brand trust in healthy foods reflects consumers’ trust in a particular brand. In the food industry, labels are relevant in generating customer confidence in the quality of the foods they consume [42,55,56,57]. Label information is a reliable indicator of the reliability and quality of food products [54,55].

Brand loyalty refers to consumers’ long-term commitment to repeat purchases from a specific brand based on the perception of higher quality and better service than any competitor [39,58,59]. Customers who demonstrate brand loyalty choose your products or services repeatedly, and they advocate on your behalf, often recommending you to friends and family [60,61]. Brand loyalty in healthy foods refers to the tendency of consumers to continue choosing a specific brand, even in the face of competitive offers or challenges [62].

The intention to purchase healthy branded foods is defined as a consumer’s predisposition to buy a healthy product [41,63,64]. According to previous studies, purchase intention significantly impacts purchasing behavior for healthy and organic foods [65,66]. Consumers’ perception of these products influences their purchase intention, ultimately translating into an actual purchase [67,68,69].

### Conceptual Model and Research Hypothesis

**Figure 1:**
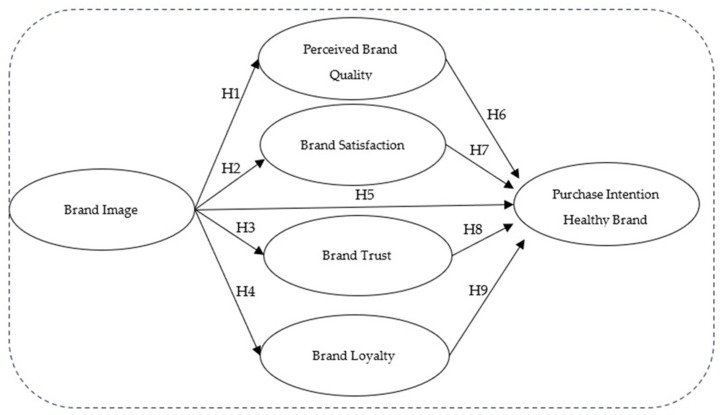
Conceptual model.

Figure 1 comprehensively overviews how different brand-related factors impact the intention to purchase healthy brand products. The figure illustrates how brand image is a crucial starting point for influencing perceived brand quality, satisfaction, trust, and loyalty. These elements and the brand image come together to determine the intention to purchase the healthy brand. This framework forms a solid basis for understanding the complexities of Peruvian consumers’ decisions regarding healthy foods, enabling brands to develop effective strategies.

Brand image strongly influences the quality perceived by the customer, which is reflected in their purchase intentions in the food industry. Also, customers are more willing to accept slightly higher prices due to their perception of food quality [44,70,71]. So, the following hypothesis can be defined as a:

**H1.**  *Brand image directly and positively impacts the perceived quality of the healthy food brand.*

The brand image of a product significantly influences customer satisfaction. When customers have a positive image of a brand, they are more likely to be satisfied with their purchases and become loyal customers [45,59,72,73,74]. For example, in a restaurant, the brand image associated with its food is a relevant predictor of customer satisfaction [59,60,72]. Given these points, the following hypothesis is proposed:

**H2.**  *Brand image directly and positively impacts healthy food brand satisfaction.*

Previous studies have indicated that consumers have low purchase intention for organic foods due to needing more trust in these products. The absence of reliability and information about these foods weakens the willingness to buy them [75,76,77]. However, if an adequate brand image was disseminated through an appropriate communication channel that provides information to the consumer, such as labels or communication campaigns, trust could be increased and, therefore, the intention to purchase healthy foods could be increased with it. Thus, the following hypothesis is proposed:

**H3.**  *Brand image directly and positively impacts trust in healthy food products.*

Brand image is an essential element in fostering consumer loyalty toward the brand [78,79,80,81]. This brand image gives companies a competitive advantage by allowing them to distinguish themselves from their rivals. Furthermore, it can generate favorable perceptions and positive emotions in consumers, often leading to choosing a particular brand over others [4,82,83]. In the food industry, recent studies show that brand image directly and positively impacts brand loyalty. Specifically, the green and healthy brand image associated with significant food chains positively influences consumer loyalty [4,84]. Consequently, it proposes the subsequent hypothesis:

**H4.**  *Brand image directly and positively impacts healthy food brand loyalty.*

A strong brand image in the healthy eating sector can significantly influence consumer behavior and decision making processes, underscoring the importance of the work performed in this field [85,86,87]. It can help a brand stand out in a competitive market and effectively communicate its commitment to health and well-being, thereby shaping the food market [19,38]. Thus, the following hypothesis is presented:

**H5.**  *Healthy food brand image has a direct and positive impact on consumer purchase intention.*

Previous studies agree that the perception of higher quality in food positively influences consumer purchase intention [43,83,88]. This perception also positively impacts the consumer’s willingness to pay more for these products ([71,89]. Considering this, the following hypothesis is proposed:

**H6.**  *The perceived quality of the healthy food brand product directly and positively impacts consumer purchase intention.*

Brand satisfaction is crucial to a positive consumer experience with a specific brand. Satisfied consumers will likely purchase and recommend it, boosting its sales and market share [39,90]. Strong satisfaction with related brands in the food industry positively impacts consumer behavior. Satisfied consumers are more likely to repeat their experience [41,42,91,92,93,94]. Subsequently, the ensuing hypothesis is proposed:

**H7.**  *Brand satisfaction with healthy food brand products directly and positively impacts consumer purchase intention.*

Previous research has confirmed a significant relationship between trust and purchasing behavior [43,95,96,97]. There is evidence that trust positively affects purchase intention by reducing the risk perceived by consumers and is considered an essential variable in the context of food consumption [98,99]. Therefore, trust in healthy food brand products directly and positively impacts consumer purchase intention [55,100]. Given these points, the following hypothesis is proposed:

**H8.**  *Brand trust in healthy food brand products directly and positively impacts consumer purchase intention.*

Brand loyalty is crucial in influencing consumer purchase intention [39,101,102]. Brand loyalty is a crucial indicator of consumer commitment to a specific brand. Consumers loyal to a brand tend to repeat their purchases and recommend the brand to others, increasing its sales and market shares [17,31,41,92,103]. In the context of healthy foods, brand loyalty may be significant. Health food consumers often look for brands they trust to provide products that align with their values and health goals. Therefore, brand loyalty can directly and positively impact purchase intention [41,42,51,103]. Consequently, the following hypothesis is offered:

**H9.**  *Brand loyalty to healthy food brand products directly and positively impacts consumer purchase intention.*

## 3. Materials and Methods

### 3.1. Sample and Procedure

Data for this quantitative study were gathered using a non-probabilistic convenience sample [104]. Data were gathered using a Google Forms online survey, with participants’ informed consent being obtained beforehand. Better coverage, quicker responses, reduced costs, shorter turnaround times, and the ability for the researcher to get in touch with the sample group are all benefits of doing online surveys [105]. Since social media users are more willing to share and reveal information, the survey was distributed through these platforms [106]. The survey was carried out during the second half of 2023 in Lima, Peru. A total of 637 people were surveyed, stating they were consumers of healthy Union brand products. The participants’ sociodemographic information is displayed in Table 1.

### 3.2. Measurements

Previously validated constructs have been used, whose items were adapted to the study context. The language of the items has been customized to shape them in the context of Union brand products and to make the language understandable to the target respondents. Consumers of the Union brand were considered because it is a brand whose value proposition is to offer healthy and nutritious products in the Peruvian market, and the study needed to ensure that the responses related to brand image and associated variables such as trust, loyalty, and satisfaction were based on the consumer’s experience. This criterion was chosen to mitigate the effects of unfamiliarity, which could confound the relationship between brand image and purchase intention.

A questionnaire adapted by Ali et al. [31] was applied to evaluate the study variables. In this way, to assess the brand image using four items, perceived quality with three items, brand trust with three items, brand loyalty with four items, and purchase intention, a two-item scale was applied. That made up a total of 17 items. Each of the questionnaire items can be seen in Appendix A. The Likert scale was used, with “1” denoting complete disagreement and “5” denoting complete agreement. There were two sections on the digital questionnaire. These 17 items were covered in the first section of the questionnaire. The second segment asked about sociodemographic information, such as age, gender, civil status, and academic level.

### 3.3. Data Analysis

Two statistical software programs were utilized to examine the data. The sociodemographic information of the subjects was examined using IBM SPSS version 22. The measurement model’s reliability, convergent validity, and discriminant validity tests were conducted using Smart-PLS version 4.0 [107]. The hypothesis was tested by the partial least squares method (PLS-SEM). With the feature of multivariate analysis, i.e., involving a number of variables equal to or greater than three, PLS-SEM is a comprehensive multivariate statistical analysis approach that combines structural and measurement components to examine the relationships between each of the variables in a conceptual model simultaneously [108]. Additionally, PLS-SEM was employed in this work since it makes theory formulation easier [109].

The path coefficients (*p*-value and t-value) were shown to be significant in assessing the structural model. The predictive relevance of the structural model was assessed using the coefficient of determination (R2). Lastly, the root mean square residual was used to assess the overall model fit (SRMR). Notably, behavioral researchers have commended PLS-SEM’s use in multidisciplinary studies [110].

## 4. Results

### 4.1. Measurement Model Evaluation

Convergent validity and construct reliability must be assessed to assess the measurement model’s internal consistency. If every indicator has a loading of more than 0.7, convergent validity is deemed appropriate [109]. The average variance extracted (AVE) and the composite reliability (CR) should be more than 0.5 [111,112]. The ideal Cronbach’s alpha coefficient is higher than 0.7. When factor-based techniques are applied, the factor typically resembles CR values [113]. Table 2 demonstrates that every loading for the 17 items in this construct had a value larger than 0.70. In the same way, all the constructions had alpha and CR values greater than 0.70, and all of the AVE values were greater than 0.50. As a result, the measurement model’s convergent validity was excellent.

The square root of each construct’s AVE was determined using the Fornell–Larcker criterion, which was used to assess discriminant validity. This square root had to be higher than the highest correlation between the construct and the other constructs in the model (126, 127). All diagonal values are more significant than the correlations, as Table 3 demonstrates. As a result, all the presumptions required to proceed with the structural model evaluation are satisfied by the measurement model.

### 4.2. Structural Model Evaluation

**Figure 2:**
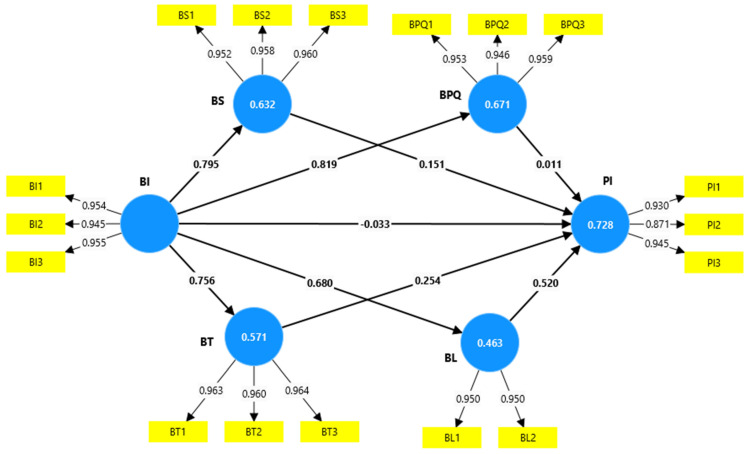
Structural model. BI: brand image; PI: purchase intention; BPQ: perceived brand quality; BS: brand satisfaction; BT: influence brand trust; BL: and brand loyalty.

The structural model was assessed using the PLS bootstrapping process with a complete result, a subsample of 5000, and a one-tailed *t*-test with a significance threshold of 0.05% following the completion of the discriminant, convergent, and reliability tests. Figure 2 displays the outcomes of the structural model with the path coefficient, which should be between −1 and +1 [107].

Chin [114] indicates that the significant, moderate, and weak measures of R are, respectively, 0.67, 0.33, and 0.19. In behavioral research, an R2 value of 0.2 is considered appropriate [115,116]. The R2 coefficients of the present work for BS, BT, BPQ, BL, and PI were 0.632, 0.571, 0.671, 0.463, and 0.728, respectively. That is, the R2 values were substantial. Therefore, the values show that the variables of the present study explain an acceptable percentage of the variance of PI. The overall fit of the model was measured using the root mean square residual (SRMR), which resulted in a value of 0.035 for this indicator, which was below the recommended threshold value of 0.080 [115], thus confirming the fit of the measurement model.

Hypothesis tests and evaluation of path coefficients can be seen in Table 4. The results show that all hypotheses were proven except H5 and H6. BI also positively and significantly influenced BPQ, BS, BT, and BL, which supports hypotheses H1, H2, H3, and H4. The results show that BS, BT, and BL positively and significantly influence PI, which supports hypotheses H7, H8, and H9. This model indicates that BI and BPQ do not influence PI, so H5 and H6 are not accepted. This result could mean that consumer preferences and priorities may differ from other markets in the Peruvian context. Peruvian consumers may prioritize factors other than brand image and perceived quality when purchasing health food. For example, health education campaigns may significantly impact purchase intent more than brand image or perceived quality. It could be that brand image is less relevant than other aspects, such as value for money or product accessibility. Also, consumers may assume that all healthy products meet a minimum quality standard, meaning that perceived quality may not act as a key differentiator.

Likewise, Table 5 shows that BT, BS, and BL are mediating variables in the relationship between BI and PI, with this mediation being direct and significant. However, BPQ is not a mediating variable in this relationship.

## 5. Discussion

The results reveal that brand image plays a fundamental role in purchasing healthy products. This is consistent with studies indicating that brand image significantly influences consumers’ perceptions, promotions, and decisions when choosing frozen foods [117]. In addition, brand image positively impacts consumer experience and overall product perception [118].

Similarly, brand image has been shown to directly and positively influence satisfaction with health food brands. The relationship between brand image and satisfaction is closely linked, especially when brand image becomes the basis for consumers’ purchase decisions and loyalty [115]. Furthermore, consumers’ perceptions of authenticity concerning health food brands underline that brand image has a direct impact on the purchase decisions regarding these products [30]

Brand image has been shown to directly influence consumer confidence in healthy food products. A strong brand image not only enhances product perception but also reinforces consumers’ confidence in choosing the product, as positive associations with the brand generate trust in the quality and authenticity of the food [116]. Furthermore, this relationship between brand image and trust has been confirmed in different contexts, including the food service sector [119] and in studies conducted in various world regions such as Asia and South America. This underlines the importance of good brand image management in strengthening trust in healthy foods.

The brand image also directly and positively impacts loyalty to health food brands. A strengthened brand image is the result of focused efforts to generate loyalty, which leads consumers to feel more committed to the brand [120]. In addition, loyal consumers are willing to pay higher prices for products that project an enhanced image, demonstrating the importance of brand perception in customers’ willingness to pay more [70]. Likewise, brand image acts as a key mediator in building loyalty; a strong brand can significantly enhance customer loyalty [121]. These aspects confirm that a strong brand image directly and positively influences brand loyalty.

It has been shown that the brand image and perceived quality of health foods does not have a significant impact on consumer purchase intention. This may be due to the fact that the purchasing of healthy products is not primarily motivated by brand image but by consumers’ personal health-related motivations [122]. Furthermore, a brand’s sustainability is not usually a determining factor in purchasing healthy and exclusive products, as consumers tend to purchase them for their health-promoting qualities rather than for the sustainable image of the brand [123]. The non-influence of brand image on purchase intention could indicate that, in the Peruvian market, brand perception is not such a determining factor for health food consumers. It could be that brand image is less relevant than other aspects, such as value for money or product accessibility. Similarly, if perceived quality also does not affect purchase intention, it may be that consumers do not consider quality as a significant differentiating factor in the health food market. It can be inferred that consumers may assume that all healthy products meet a minimum standard of quality, meaning that perceived quality may not be a key differentiator.

Consumer satisfaction with a brand has been shown to be a crucial factor when purchasing a brand’s products or services. Brand satisfaction directly influences purchase intention, as a positive brand experience fosters long-term relationships with consumers, which in turn drives their purchase intention [20,124,125]. In this sense, it can be stated that brand satisfaction positively impacts consumers’ purchase decisions.

Another key variable is brand trust in health food products, which also directly and positively affects consumer purchase intention. Brand trust has a greater effect on purchase intention than on relational commitment, indicating that increasing brand trust can increase purchase intention [126]. This trust is based on consumers’ perception of brand trustworthiness, which significantly impacts their purchase decisions [127]. In addition, brand awareness and brand image have been found to significantly influence consumers’ trust in a brand. In the case of organic food in Brazil, surprisingly, it was found that only emotional value motivates purchase intention [128], which demonstrates a different behavior than expected in this market. Despite this difference, it can be concluded that brand trust has a direct impact on consumer purchases.

Finally, loyalty to a health food brand also directly and positively influences purchase intention. There is a clear relationship between brand loyalty and brand trust, as demonstrated by a study that found that 76% of loyalty-motivated purchase behaviors are based on brand trust [129]. In the case of organic food, it has been concluded that perceived quality and brand loyalty are key factors that positively affect the purchase decision, while brand awareness and brand associations do not have a significant effect [130]. From the analysis of brand loyalty, it can be concluded that this variable positively influences consumers’ purchase intentions.

### 5.1. Theoretical and Practical Implications

The study of the purchase intention of healthy foods and the determining role of brand image in a developing country’s market has several theoretical and practical implications. Theoretically, this study contributes to consumer behavior and healthy food marketing literature. It offers a deeper understanding of how a brand image can influence purchase intention regarding nutritious foods in a developing country context. In addition, it opens new avenues of research in food marketing and consumer behavior, focusing on topics such as brand personality. This would provide new insight into how consumers perceive and relate to healthy food brands. From a practical perspective, this study’s findings are informative and actionable actions. They can empower marketers and decision-makers in the health food industry with knowledge regarding how to develop more effective brand strategies, thereby increasing demand for their healthy products.

### 5.2. Management Implications

The implications for managing healthy food purchase intention and the role of brand image are significant. Managers should consider the importance of brand image when developing marketing strategies to increase customer preferences for healthy foods.

First, managers must invest in building a strong and positive brand image. This can be achieved through various strategies such as advertising, public relations, sponsoring health-related events, and creating brand ambassadors. This way, consumers will perceive these foods as being better for their health and lifestyle. Therefore, managers must continually monitor brand image perception among consumers and adjust their marketing strategies according to market changes. In other words, companies selling healthy foods in Peru may need to review their marketing strategies. If brand image and perceived quality are not decisive factors, they could focus on other aspects, such as price, distribution, or awareness campaigns about the benefits of healthy foods.

In addition, managers must consider the market’s specific characteristics in a developing country. These include factors such as the level of health awareness, local taste preferences, and consumers’ purchasing power. This way, a preference for healthy foods can be created rather than rejected.

Educational campaigns informing consumers about the benefits of products and how to distinguish high-quality products may be useful, as this could influence purchase intention more than brand perception.

Finally, the Ministry of Health and the Ministry of Education should play a pivotal role in promoting healthy lifestyles. Their commitment to practical courses, such as healthy eating and lifestyle programs, can inspire and motivate new generations to have a broader vision and prefer healthy food brands.

## 6. Conclusions

Based on the study’s results and response to the research question “What is the relationship between BI and PI of healthy foods?”, it is concluded that these two variables have no significant correlation. Therefore, it is proven that brand image does not directly affect purchase intention, which represents that the perception and assessment of BI in the context of healthy foods is not as significant a determining element as other factors, such as quality, satisfaction, trust, and loyalty towards the product.

In turn, the study answered the question of how BI influences BPQ, BS, BT, and BL, proving that, in the context of healthy foods, brand image (BI) has a significant impact on brand perceived quality (BPQ), brand satisfaction (BS), brand trust (BT), and brand loyalty (BL). It is possible to affirm that brand image is an integral indicator of consumer expectations and experiences. A positive brand image reinforces the perception of quality, where consumers assume that a well-positioned brand offers high-quality products. In this sense, a well-perceived brand satisfies consumers by delivering on its promises and building trust, as consumers associate the brand with reliability and consistency in product quality. In turn, it fosters long-term loyalty, as consumers who trust and are satisfied with a brand are likelier to continue choosing it.

However, it does have a significant impact on brand satisfaction (BS), brand trust (BT), and brand loyalty (BL). This result can be explained by the fact that, although consumers may recognize the high quality of a product, this does not always translate into a purchase intention due to the barriers that prevent a purchase. On the other hand, customer satisfaction encourages the consumer to perceive the product as being adequate and of sufficient quality to meet consumer expectations. Furthermore, a positive perception of quality strengthens BL, as consumers believe the brand will continue providing high-quality products. This trust, in turn, fosters long-term loyalty, as consumers prefer to continue with a brand that has proven to be reliable and of high quality.

### 6.1. Limitations of the Study

Despite the valuable contributions of this study to understanding the role of brand image in purchase intention for healthy foods in a developing country, some limitations have been recognized. First, using a cross-sectional study design limits the ability to establish definitive causal relationships between the variables studied. The observed correlations between brand image, perceived quality, satisfaction, trust, loyalty, and purchase intention provide a valuable snapshot but do not allow us to infer causality or the effect of these relationships over time.

Secondly, the sample used in this study was obtained through non-probabilistic convenience sampling, which may introduce selection biases and limit the generalization of the results. Although the survey was widely distributed through social media, participants may not represent the general population of healthy food consumers in the Peruvian context.

Additionally, the study focused exclusively on the city of Lima, which may not fully reflect the attitudes and behaviors of consumers in other regions of the country. Cultural, economic, and social differences between regions can influence the perception of brand image and the intention to purchase healthy products.

Another significant limitation of this study is the inclusion criterion, which required participants to have prior experience with Union brand products. This could have introduced a selection bias, as it potentially excluded a portion of the consumer market who may have different perceptions based on indirect knowledge of the brand.

Finally, the research was based on self-report through questionnaires, which may be subject to social desirability biases and measurement errors. Although validated and reliable scales were used, the subjective nature of participants’ perceptions could have affected the accuracy of the data collected.

### 6.2. Future Investigations

Some recommendations for future research address these shortcomings and increase the body of knowledge on the subject. First, longitudinal studies should be carried out to evaluate how the relationships between brand image, perceived quality, satisfaction, trust, loyalty, and purchase intention evolve over time. This would allow for a deeper understanding of these relationships’ causal dynamics and stability in the context of healthy foods.

Likewise, future research could benefit from implementing experimental designs that allow for manipulating the brand image and observing its direct and indirect effects on the dependent variables. This would help establish robust causal relationships and identify the underlying mechanisms explaining how and why brand image influences purchase intention.

A replication of this study in diverse cultural contexts and across the nation would enhance the representativeness and generalizability of the findings. Comparing the results between different regions could reveal significant variations in consumer perceptions and behaviors, providing a more complete and nuanced view of the healthy food market in Peru.

Further future research should consider including a broader sample of participants, including those with varying degrees of familiarity with the brand, to assess how initial exposure to the brand influences the observed relationships.

Additionally, future research could explore other factors influencing purchase intention regarding healthy foods, such as the influence of marketing campaigns, product characteristics (e.g., ingredients, labeling), and health and wellness trends in the market and throughout society, including additional variables that could provide a more comprehensive understanding of the determinants of purchase intention.

Complementary data collection methods, such as in-depth interviews or focus groups, are recommended for obtaining a qualitative perspective that enriches the quantitative findings. These methods could help identify underlying motivations, barriers, and facilitators of purchase intention that questionnaires cannot fully capture.

Additional qualitative research, such as interviews or focus groups, could provide a deeper understanding of the factors affecting purchase intention in the Peruvian context. This may help to identify the motivations and barriers consumers face when choosing healthy products.

This study provides important insights into the role of brand image in the purchase intention of healthy foods in a developing context; future research should address its limitations through longitudinal, experimental designs and more diverse and representative methodological approaches. This will allow us to advance theoretical and practical knowledge, contributing to developing more effective marketing strategies adapted to consumers’ needs and preferences in emerging markets.

## Figures and Tables

**Table 1:** Sociodemographic data of the participants.

Variables	Categories	Frequencies	%
Gender	Man	222	34.9
	Women	415	65.1
Age	18–20 years	290	45.5
	21–25 years	254	39.9
	26–30 years	45	7.1
	31–35 years	24	3.8
	35 to 40 years	8	1.3
	More than 40 years	16	2.5
Academic level	University (postgraduate)	67	10.5
	University (undergraduate)	570	89.5
Civil status	Married	35	5.5
	Cohabitant	2	0.3
	Divorced	5	0.8
	Single	595	93.4

**Table 2:** Validation of the measurement model (reliability and convergent validity).

Construct	Item	Loading	Alpha	CR	AVE
Brand Image (BI)	BI1	0.954	0.948	0.966	0.905
	BI2	0.945			
	BI3	0.955			
Brand Loyalty (BL)	BL1	0.950	0.896	0.949	0.902
	BL2	0.950			
Brand Perceived Quality (BPQ)	BPQ1	0.953	0.949	0.967	0.908
	BPQ2	0.946			
	BPQ3	0.959			
Brand Satisfaction (BS)	BS1	0.952	0.953	0.970	0.915
	BS2	0.958			
	BS3	0.960			
Brand Trust (BT)	BT1	0.963	0.960	0.974	0.926
	BT2	0.960			
	BT3	0.964			
Purchase Intention (PI)	PI1	0.930	0.904	0.940	0.839
	PI2	0.871			
	PI3	0.945			

**Table 3:** Validity discriminant (Fornell–Larcker criterion).

	BI	BL	BPQ	BS	BT	PI
Brand Image (BI)	0.951					
Brand Loyalty (BL)	0.680	0.950				
Brand Perceived Quality (BPQ)	0.819	0.765	0.953			
Brand Satisfaction (BS)	0.795	0.758	0.813	0.956		
Brand Trust (BT)	0.756	0.814	0.816	0.822	0.962	
Purchase Intention (PI)	0.642	0.827	0.712	0.737	0.786	0.916

**Table 4:** PLS path model main effects.

Hypothesis		Beta	Standard Deviation	t	*p*-Value	Decision
H1	BI → BPQ	0.819	0.017	48.259	0.000	Supported
H2	BI → BS	0.795	0.022	36.269	0.000	Supported
H3	BI → BT	0.756	0.022	34.563	0.000	Supported
H4	BI → BL	0.680	0.028	24.264	0.000	Supported
H5	BI → PI	−0.033	0.045	0.732	0.464	Rejected
H6	BPQ → PI	0.011	0.060	0.180	0.857	Rejected
H7	BS → PI	0.151	0.054	2.791	0.005	Supported
H8	BT → PI	0.254	0.060	4.249	0.000	Supported
H9	BL → PI	0.520	0.055	9.513	0.000	Supported

**Table 5:** Mediation effect.

	Original Sample	Standard Deviation	t	*p*-Values
BI → BT → PI	0.192	0.045	4.229	0.000
BI → BS → PI	0.120	0.043	2.790	0.005
BI → BPQ → PI	0.009	0.049	0.180	0.857
BI → BL → PI	0.354	0.040	8.883	0.000

## Data Availability

The original contributions presented in the study are included in the article material, further inquiries can be directed to the corresponding author.

## Appendix A. Questionnaire

**Variable**	**CODE**	**Items**
Brand Image (BI)	BI1	The Union brand has a good reputation
	BI2	The Union brand addresses my health concerns
	BI3	The Union brand is reliable
Brand Loyalty (BL)	BL1	Personally, I prefer the Union brand to other health brands
	BL2	Personally, I recommend the Union brand to my friends and family
Brand Perceived Quality (BPQ)	BPQ1	The quality of the Union brand is very good
	BPQ2	The quality of the Union brand is maintained over time.
	BPQ3	The quality of the Union brand is reliable
Brand satisfaction (BS)	BS1	I am personally happy with my decision to choose the Union brand because of its commitment to healthy living.
	BS2	I personally believe that the Union brand is the right thing to buy because of its guarantee
	BS3	Overall, I am satisfied with the Union brand because of my health concerns.
Brand Trust (BT)	BT1	Personally, I believe the Union brand is safe
	BT2	Personally, I believe the Union brand is honest
	BT3	Personally, I believe the Union brand is trustworthy
Purchase Intention (PI)	PI1	Personally, I intend to buy Union brand products upon my next purchase
	PI2	Personally, I intend to pay even a little more for the Union brand
	PI3	Personally, I will choose Union brand products whenever I need to buy healthy products.
